# Trauma levels and perspectives on dignified death among nurses and physicians who directly experienced the recent earthquake

**DOI:** 10.1371/journal.pone.0311184

**Published:** 2024-10-24

**Authors:** Gamze Özbek Güven, Mehmet Karataş, Sibel Kaynak

**Affiliations:** 1 Yuksek Ihtisas University Faculty of Medicine, Department of Medical History and Ethics, Ankara, Türkiye; 2 Inonu University Faculty of Medicine, Department of Medical History and Ethics, Malatya, Türkiye; 3 Malatya Doğanşehir Şehit Esra Köse Başaran State Hospital, Malatya, Türkiye; University of Nicosia, NEPAL

## Abstract

**Background:**

Unexpected, sudden, and tragic losses can prompt us to reflect on the concept of a "good death." The earthquake disaster that struck our country in 2023 vividly demonstrated the challenging impact of such events, which can turn lives upside down and compel us to question the notion of a "good death." This study aims to determine the perceptions of a "good death" and the levels of trauma experienced by physicians and nurses who directly witnessed the earthquake disaster, and to understand the relationship between these factors.

**Methods:**

This cross-sectional study was conducted between October 1 and December 31, 2023, using phone interviews facilitated by a web-based Google form. Data were collected from 560 healthcare professionals (280 nurses and 280 doctors) working in hospitals located in Kahramanmaraş, Hatay, Malatya, and Adıyaman provinces in Türkiye, which were directly impacted by the earthquake of February 6, 2023, and who consented to participate in the study. Trauma levels were assessed using the "Post-Earthquake Trauma Level Determination Scale," and perceptions of a good death were evaluated using the "Good Death Scale (GDS)." Data were analyzed using SPSS 25 and AMOS 24 software. Normal distribution was checked with the Kolmogorov-Smirnov Test. Independent t-tests were used to compare independent binary groups, Pearson correlation analysis was used to examine the relationship between scale scores, and Cronbach’s α coefficient was used to evaluate the reliability of the scales (Good Death Scale: 0.931; Trauma Scale: 0.957). Structural equation modeling and multi-group analysis were conducted to examine the relationship between scale scores according to the profession variable.

**Findings:**

The mean score for the perception of a good death was found to be 52.76 ± 8.77 for physicians and 55.84 ± 9.63 for nurses. A statistically significant difference was detected between physicians and nurses in the "psychosocial spirituality," "personal control," and "clinical" sub-dimensions of the scale (p<0.05). The mean trauma scores were 56.81 ± 17.58 for physicians and 64.82 ± 18.56 for nurses. A significant difference was found in the trauma scale and its sub-dimensions ("excitement limitation," "emotional," "cognitive restructuring," "sleep problems") (p<0.05). It was observed that higher trauma levels positively influenced good death perception scores.

**Conclusion:**

This study reveals that healthcare workers are deeply affected psychologically by major disasters, with high levels of trauma. A significant relationship was found between trauma levels and perceptions of a good death. These findings provide an important basis for future research to understand how trauma shapes the lives and job performance of healthcare workers in the long term.

## Introduction

Death is an inevitable and profoundly impactful aspect of human existence. The advancement of modern medicine and the expansion of healthcare services have led to an extension of life, thereby postponing the process of dying [1]. Along with this extension, the expansion of end-of-life care services has increased interest in the concept of a ’good death,’ leading to a more comprehensive examination of this topic from various perspectives [2].There is no clear definition of what constitutes a good death or what characteristics the term encompasses. Terms such as "good death," "peaceful death," "appropriate death," "desired death," and "dignified death" are sometimes used interchangeably and sometimes convey different meanings [3]. Generally, a good death includes elements such as a painless death (not suffering from life-support devices, dying with care), a natural death (death at the end of life, death due to illness or disease), and a death free of anxiety (preparation for death, spiritual and faith practices, management of family and property before death, dying among family members and in familiar surroundings) [4–7].

The concept of a "good death" is dynamic and multifaceted; it is influenced by the values, beliefs, and cultural structures of societies and changes over time [8,9]. In Western culture, a good death is generally understood as a "comfortable death, acceptance of death, and not being a burden for care," while in Asian culture, it is defined as "meeting care and spiritual needs until the very end" [7]. A study conducted in East Asia revealed that for Japanese physicians, a good death is associated with physical comfort and autonomy; for Taiwanese physicians, it involves the completion of life and liberation from devices; and for Korean physicians, it is characterized by cognitive integrity [10].

Death, takes on a different dimension in disasters such as earthquakes, where thousands of people die suddenly and without preparation, deeply affecting perceptions of death. On February 6, 2023, a 7.8 magnitude earthquake struck southeastern Türkiye near the northern border of Syria. Türkiye is a country with a high probability of earthquakes and has experienced various earthquakes throughout its history. However, this earthquake occurred with unprecedented intensity. It simultaneously affected many cities and caused massive destruction [11]. While the shock of the first earthquake persisted, a second earthquake struck. This disaster led to the collapse of thousands of buildings, including hospitals, leaving people trapped under the rubble waiting for rescue. Harsh weather conditions hindered the rapid arrival of search and rescue and first aid teams to the region. Over time, hopes of being rescued from the rubble diminished, and the number of casualties increased. In 2023, natural disasters worldwide caused the deaths of at least 95,000 people. Of these deaths, 62% were linked to the earthquakes in Türkiye and Syria. These earthquakes have been recorded as the deadliest disaster since the 2010 Haiti earthquake [12].

This deadly disaster has revealed the challenging impacts of a death that suddenly changes lives. Although the general belief system in our country tends to view concepts such as euthanasia and peaceful death negatively, the experiences and impacts of this extraordinary process may influence our perceptions of a good death.

Disasters cause profound psychological effects and lasting emotional scars on individuals and communities. However, often overlooked is the fact that rescue workers involved in disaster response efforts also face significant physical and psychological challenges [13]. The earthquake disaster in our country not only caused health professionals to experience the earthquake personally and suffer as victims but also led them to witness many tragic events while making intensive efforts to save other people’s lives. These challenging experiences can significantly affect the trauma levels and perspectives on death of health professionals.

The aim of this study is to determine the levels of trauma experienced by physicians and nurses who directly experienced the earthquake and their perceptions of a good death, and to understand the relationship between these factors. Evaluating the perceptions of a good death among health professionals who were trying to save lives during the earthquake can strengthen their ability to cope with these situations and improve their communication with patients. It can also help them understand their personal traumas and manage the impacts on their professional lives.

This approach represents an important step in providing support and resources to health professionals and can contribute to the development of training programs for other healthcare workers who might encounter similar situations.

## Materials and methods

This study was conducted as a questionnaire based research among physicians and nurses who directly experienced and were affected by the earthquake. The questionnaire forms included appropriate scales to assess the participants’ levels of trauma and their views on the concept of a good death. The survey results were evaluated using quantitative data analysis methods, leading to the study findings.

### Sample

This cross-sectional study was conducted among physicians and nurses who experienced the earthquake in the provinces of Kahramanmaraş, Hatay, Malatya, and Adıyaman in Türkiye. The cross-sectional method was chosen due to its efficiency in collecting data at a single point in time to examine the status and relationships among the variables of interest [14]. The research using the simple random sampling method was conducted over a three-month period from October to December 2023. The study was designed in accordance with the STROBE guidelines for cross-sectional research, and the data were collected using Google Forms, a free online form creation tool.

During the earthquake, hospital administrations created phone communication groups to facilitate easy and collective communication with healthcare personnel. In this study, participants were reached through these groups with permission from the hospital administrations. Participants were informed about the purpose of the study, that participation was voluntary, that they could withdraw at any time, and how to complete the survey. Those who agreed to fill out the form first gave their consent and then answered the questions. The anonymity and confidentiality of the participants’ information were ensured. The study was concluded once the targeted sample size was reached. Individual information was kept confidential and secure. The data were anonymized and stored in encrypted databases, accessible only to the researchers involved in the study.

The sample size was calculated using a well-known sampling method (95% confidence interval, alpha = 0.05, p = 0.50, N = 572). As a result of these calculations, it was determined that at least 231 nurses and physicinas needed to be included in the study. The inclusion criteria were as follows: (1) being 18 years or older, (2) living and working in the earthquake zone, (3) experiencing the earthquake moment, (4) willingness to participate in the study, and (5) completing the survey in full. A total of 280 physicians and 280 nurses who met these criteria were included in the study (n = 560).

### Data collection ınstruments

Data were collected using the Information Form, the Post-Earthquake Trauma Level Determination Scale, and the Good Death Scale. The scales are in the participants’ native language.

#### Physician and nurse ınformation form

This form was designed by the researchers based on the literature. It includes questions about socio-demographic characteristics (such as age, gender, marital status, years of work experience), death experience, frequency of thinking about death, and the status of receiving education about death.

#### Post-earthquake trauma level determination scale

Developed by Tanhan and Kayri (2013), this scale aims to measure trauma symptoms that may arise in individuals after an earthquake [15]. The scale consists of 20 items and 5 dimensions. According to the analysis results for determining the construct validity of the scale, the model fit indices were calculated as RMSEA = .000, GFI = .94, AGFI = .92, NFI = .88, RMR = 0.08. The internal consistency coefficient calculated to determine the reliability of the scale is 0.87. A score range of 52.385 ± 5.051 corresponds to a threshold value indicating that individuals are traumatized. Scores above or below this value indicate a high or low level of post-earthquake traumatic symptoms, respectively.

#### Good Death Scale (GDS)

Developed by Schwartz et al. in 2003, was validated and tested for reliability in Turkish by Fadıloğlu and Aksu [16,17]. It is a four-point Likert-type scale (1 = not important, 2 = somewhat important, 3 = important, 4 = very important) consisting of 17 questions and three sub-dimensions. These three sub-dimensions are named "psychosocial and spiritual sub-dimension," "personal control sub-dimension," and "clinical sub-dimension." The psychosocial and spiritual sub-dimension consists of 9 questions (4, 6, 7, 8, 9, 10, 11, 12, 13); the personal control sub-dimension consists of three questions (15, 16, 17); and the clinical sub-dimension consists of five questions (1, 2, 3, 5, 14). Scale values can range from 17 to 68, with a higher value indicating a greater importance placed on the concept of a good death.

The Good Death Scale provides a comprehensive assessment by encompassing three key areas. High scores among these areas indicate that a specific sub-area of the Good Death Concept is prominent. The first section, Psychosocial and Spiritual Sub-dimension, reflects a good death in terms of psychosocial or spiritual aspects, relating to beliefs in an afterlife, meaningful social support, and spiritual beliefs and practices (such as worship). The second section, Personal Control Sub-dimension, emphasizes personal control, focusing more on the physical aspects of the death experience. This section is related to negative attitudes and mood symptoms and does not show connections with spiritual or belief-related factors. The third section, Clinical Sub-dimension, addresses a good death from a clinical and biomedical perspective, associating death with the perspective of escaping the negative aspects of worldly life [16].

### Ethical approval

This study was reviewed and approved by the Non-Interventional Ethics Committee of Malatya Turgut Ozal University (Protocol code: 14.09.2023/34).

### Statistical analysis

The statistical analysis of the study was performed using SPSS (Statistical Program for Social Sciences) version 25 and AMOS version 24. The significance level (p) for comparison tests was set at 0.05. Data values were expressed as frequency, percentage, mean, and standard deviation. The normal distribution of the data was checked using the Kolmogorov-Smirnov Test. Since the data were normally distributed (p> 0.05), parametric test methods were used for further analysis. Comparisons between independent groups were conducted using the independent t-test, as the normality assumption was met. Pearson correlation analysis was used to compare scale scores, and Cronbach’s alpha coefficient was used to evaluate the reliability of the scales. The Cronbach’s alpha coefficient was found to be 0.931 for the Good Death Scale and 0.957 for the Trauma Scale.

To conduct multivariate analysis, data were first checked for multivariate normal distribution and multicollinearity. A Structural Equation Modeling (SEM) was established, with the trauma level scale score as the independent variable and the good death scale score as the dependent variable. To examine whether there were changes in the relationship between the scale scores according to the profession (physician and nurse), each group was coded and multi-group analysis was applied to the paths between the scales. In this correlational study, the most preferred analysis method, Structural Equation Modeling, was used [18]. In the study, the "Observations farthest from the centroid (Mahalonobis Distance) Menu" in the AMOS program was used for the multivariate normal distribution control for multivariate analysis, and Marida’s coefficient was found to be 3.906 [18]. A calculated value below 8 indicates that the data are suitable for multivariate normal distribution [19]. VIF (variance influence factor) was used for the multiple linear connection analysis and the value was calculated below 5.

## Results

A total of 560 individuals, including 280 physicians and 280 nurses, participated in the study. Among the physicians, 60% were women, while 77.1% of the nurses were women; 40% of the physicians and 22.9% of the nurses were men. In terms of age, 53.6% of the physicians and 63.9% of the nurses were between 25–34 years old. Regarding marital status, 56.1% of the physicians were married, and 55% of the nurses were single.

In terms of work experience, 55% of the physicians and 62.1% of the nurses had 0–5 years of experience. Concerning housing, 47.1% of the physicians and 51.1% of the nurses lived in slightly damaged houses. Additionally, 81.1% of the physicians and 82.9% of the nurses did not lose any first-degree relatives during the earthquake.

A significant majority, 90.4% of the physicians and 92.1% of the nurses, were still working in the earthquake-affected areas. Finally, 25.7% of the physicians and 33.2% of the nurses reported that they often thought about death.

No statistically significant difference was found between physicians and nurses regarding variables such as home damage, loss of a relative in the earthquake, and being in the earthquake zone (p>0.05). However, a statistically significant difference was found in terms of the frequency of thinking about death (p<0.05). Nurses reported thinking about death more frequently (Table 1).

**Table 1:** Comparison of demographic ınformation according to groups.

Variable	Grup	n / %	Profession		Total	χ^2^	p
			Physician	Nurse			
**Gender**	**Female**	**n**	168	216	384	***18*.*304***	***0*.*001*** *
		**%**	**60%**	**77.1%**	68.6%		
	**Male**	**n**	112	64	176		
		**%**	**40.0%**	**22.9%**	31.4%		
**Age**	**18–24**	**n**	24	49	73	***32*.*571***	***0*.*001*** *
		**%**	8.6%	17.5%	13.0%		
	**25–34**	**n**	150	179	329		
		**%**	**53.6%**	**63.9%**	58.8%		
	**35–44**	**n**	62	37	99		
		**%**	22.1%	13.2%	17.7%		
	**45 years and above**	**n**	44	15	59		
		**%**	15.7%	5.4%	10.5%		
**Marital satatus**	**Single**	**n**	123	154	277	***6*.*429***	***0*.*011*** *
		**%**	43.9%	**55.0%**	49.5%		
	**Married**	**n**	157	126	283		
		**%**	**56.1%**	45.0%	50.5%		
**Working experience**	**0–5 years**	**n**	154	174	328	***18*.*097***	***0*.*001*** *
		**%**	**55.0%**	**62.1%**	58.6%		
	**6–15 years**	**n**	49	53	102		
		**%**	17.5%	18.9%	18.2%		
	**16–20 years**	**n**	12	23	35		
		**%**	4.3%	8.2%	6.3%		
	**21 year and above**	**n**	65	30	95		
		**%**	23.2%	10.7%	17.0%		
**House damage**	**Undamaged**	**n**	45	42	87	6.625	0.157
		**%**	16.1%	15.0%	15.5%		
	**Little damaged**	**n**	132	143	275		
		**%**	**47.1%**	**51.1%**	49.1%		
	**Middle damaged**	**n**	35	28	63		
		**%**	12.5%	10.0%	11.3%		
	**Heavily damaged**	**n**	44	55	99		
		**%**	15.7%	19.6%	17.7%		
	**Destroyed**	**n**	24	12	36		
		**%**	8.6%	4.3%	6.4%		
**Death of relative**	**No**	**n**	227	232	459	0.193	0.660
		**%**	**81.1%**	**82.9%**	82.0%		
	**Yes**	**n**	53	48	101		
		**%**	18.9%	17.1%	18.0%		
**Still working in the earthquake zone**	**No**	**n**	27	22	49	0.358	0.550
		**%**	9.6%	7.9%	8.8%		
	**Yes**	**n**	253	258	511		
		**%**	**90.4%**	**92.1%**	91.3%		
**Thought about death**	**None**	**n**	36	17	53	***22*.*450***	***0*.*001*** *
		**%**	12.9%	6.1%	9.5%		
	**Now and again**	**n**	38	25	63		
		**%**	13.6%	8.9%	11.3%		
	**Sometimes**	**n**	59	61	120		
		**%**	21.1%	21.8%	21.4%		
	**Often**	**n**	72	93	165		
		**%**	25.7%	33.2%	29.5%		
	**Increasingly**	**n**	46	31	77		
		**%**	16.4%	11.1%	13.8%		
	**Always**	**n**	29	53	82		
		**%**	10.4%	18.9%	14.6%		

*p<0.05; There is a statistically significant difference between the groups.

The average good death score was determined to be 52.76 ± 8.77 for physicians and 55.84 ± 9.63 for nurses. According to this result, the perception of a good death was found to be high for both physicians and nurses. Additionally, a statistically significant difference was found between physicians and nurses in terms of "psychosocial spirituality", "personal control", and "clinical" scores (p<0.05). It was determined that nurses are more sensitive compared to physicians (Table 2).

**Table 2:** Comparison of scale scores according to groups.

Variables	Groups	Mean ± sd	t value	p value
**Psychosocial Spiritual**	**Physician**	27.65 ± 4.95	***-4*.*395***	***0*.*001*** *
	**Nurse**	29.58 ± 5.46		
**Self Control**	**Physician**	9.86 ± 1.88	***-2*.*323***	***0*.*021*** *
	**Nurse**	10.25 ± 2.12		
**Clinic**	**Physician**	15.25 ± 3.02	***-2*.*845***	***0*.*005*** *
	**Nurse**	16 ± 3.21		
**Good Death**	**Physician**	52.76 ± 8.77	***-3*.*955***	***0*.*001*** *
	**Nurse**	55.84 ± 9.63		
**Behavior Problems**	**Physician**	10.34 ± 3.85	***-5*.*289***	***0*.*001*** *
	**Nurse**	12.11 ± 4.08		
**Excitement Limitation**	**Physician**	13.34 ± 4.84	***-5*.*171***	***0*.*001*** *
	**Nurse**	15.63 ± 5.62		
**Affective**	**Physician**	11.16 ± 3.76	***-3*.*744***	***0*.*001*** *
	**Nurse**	12.38 ± 3.93		
**Cognitive Structuring**	**Physician**	13.7 ± 4.19	***-3*.*933***	***0*.*001*** *
	**Nurse**	15.2 ± 4.81		
**Sleep Problems**	**Physician**	8.27 ± 3.43	***-4*.*12***	***0*.*001*** *
	**Nurse**	9.5 ± 3.61		
**Trauma**	**Physician**	56.81 ± 17.58	***-5*.*241***	***0*.*001*** *
	**Nurse**	64.82 ± 18.56		

sd; standart deviation.

*p<0.05; There is a statistically significant difference between the ***groups***.

The average trauma scores were determined to be 56.81 ± 17.58 for physicians and 64.82 ± 18.56 for nurses. A statistically significant difference was found between physicians and nurses in terms of the trauma scale and its subdimensions, "excitement limitation", "emotional", "cognitive restructuring" and "sleep problems" scores (p<0.05). It was determined that nurses were more affected compared to physicians (Table 2).

There is a statistically significant positive correlation between the Good Death Scale and its subdimensions, "psychosocial spirituality", "personal control", "clinical" scores, and the Trauma Scale and its subdimensions, "excitement limitation", "emotional", "cognitive restructuring", and "sleep problems" scores (p<0.05) (Table 3).

**Table 3:** Relationships between scale scores.

Scores	Value	Self Control	Clinical	Good Death	Behavior Problems	Excitement Limitation	Affective	Cognitive Structuring	Sleep Problems	Trauma
**Psychosocial Spiritual**	r	0.669	0.731	0.958	0.328	0.217	0.397	0.363	0.285	0.363
	p	0.001*	0.001*	0.001*	0.001*	0.001*	0.001*	0.001*	0.001*	0.001*
**Personal Control**	r		0.522	0.771	0.275	0.179	0.294	0.236	0.187	0.269
	p		0.001*	0.001*	0.001*	0.001*	0.001*	0.001*	0.001*	0.001*
**Clinical**	r			0.864	0.338	0.313	0.353	0.362	0.313	0.389
	p			0.001*	0.001*	0.001*	0.001*	0.001*	0.001*	0.001*
**Good Death**	r				0.359	0.267	0.407	0.379	0.307	0.395
	p				0.001*	0.001*	0.001*	0.001*	0.001*	0.001*
**Behavior Problems**	r					0.724	0.652	0.701	0.713	0.877
	p					0.001*	0.001*	0.001*	0.001*	0.001*
**Excitement Limitation**	r						0.650	0.670	0.645	0.876
	p						0.001*	0.001*	0.001*	0.001*
**Affective**	r							0.724	0.609	0.838
	p							0.001*	0.001*	0.001*
**Cognitive Structuring**	r								0.673	0.878
	p								0.001*	0.001*
**Sleep Problems**	r									0.831
	p									0.001*

r; pearson correlation coefficient

*p<0.05; There is a statistically significant relationship between the variables.

## The relationship between the concept of a good death and trauma level

**Fig 1:**
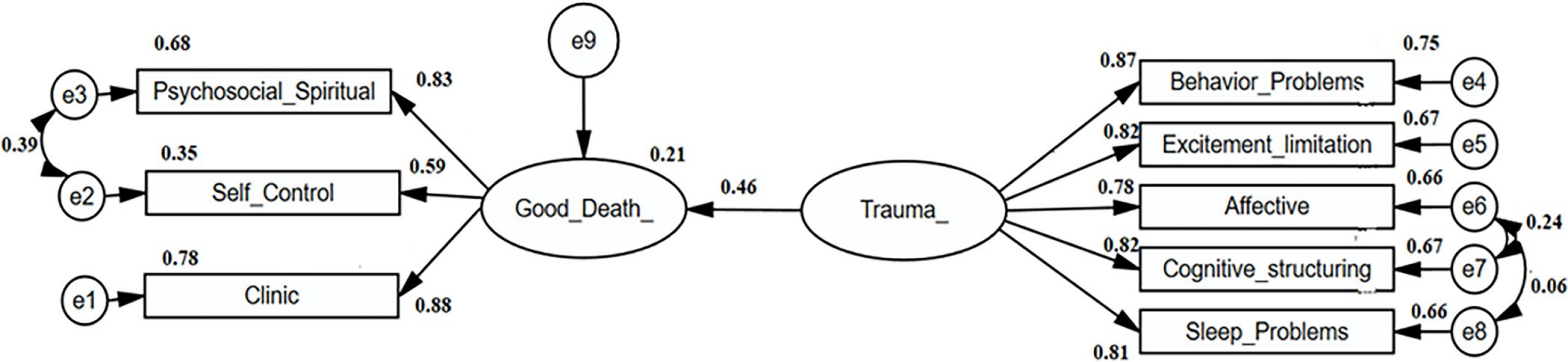
SEM diagram of the relationship between good death concept and trauma level. The figure shows the modeling study (GLM) illustrating the relationship between trauma level and the concept of a good death. The model showed that increasing trauma levels positively affected good death scores. In other words individuals who experienced higher levels of trauma had higher good death scores.

In the study, the effect of trauma level on the perception of a "good death" was examined. The modeling study (SEM) mathematically expressed the relationship between the trauma level and the concept of a good death (Fig 1).

According to the model results, each one-point increase in trauma level increases the good death score by an average of 0.57 points. The trauma level explains 21% of the variation in good death scores. The model’s fit was confirmed by various statistical criteria. Indices such as RMSEA, GFI, IFI, and CFI used for model fit indicated that the model fit the data well (Table 4).

**Table 4:** Regression coefficients for the relationship between good death concept and trauma level.

**Independent**	**Dependent**	**β**_**1**_	**β**_**2**_	**p**	**R** ^**2**^
**Trauma Level**	**Good Death**	0.46	0.57	0.001*	0.21

β_1_; Standardized regression coefficents, β_2_; Unstandardized regression coefficients

*p<0,05; t test result for the significance of the regression coefficients, R^2^; Explanatory coefficients.

The fit indices of the established SEM were examined and it was found that the model is statistically significant. The value of χ2/df was calculated as 4.460. The sample size used to establish the model was determined to be sufficient, with RMSEA (Root Mean Square Error of Approximation) calculated at 0.07, GFI (Goodness of Fit Index) at 0.971, IFI (Incremental Fit Index) at 0.980, and CFI (Comparative Fit Index) at 0.981. Since the relationship established between the scales and the SEM was found to be statistically significant and adequate, the analysis proceeded to the multiple group analysis stage [20].

**Fig 2:**
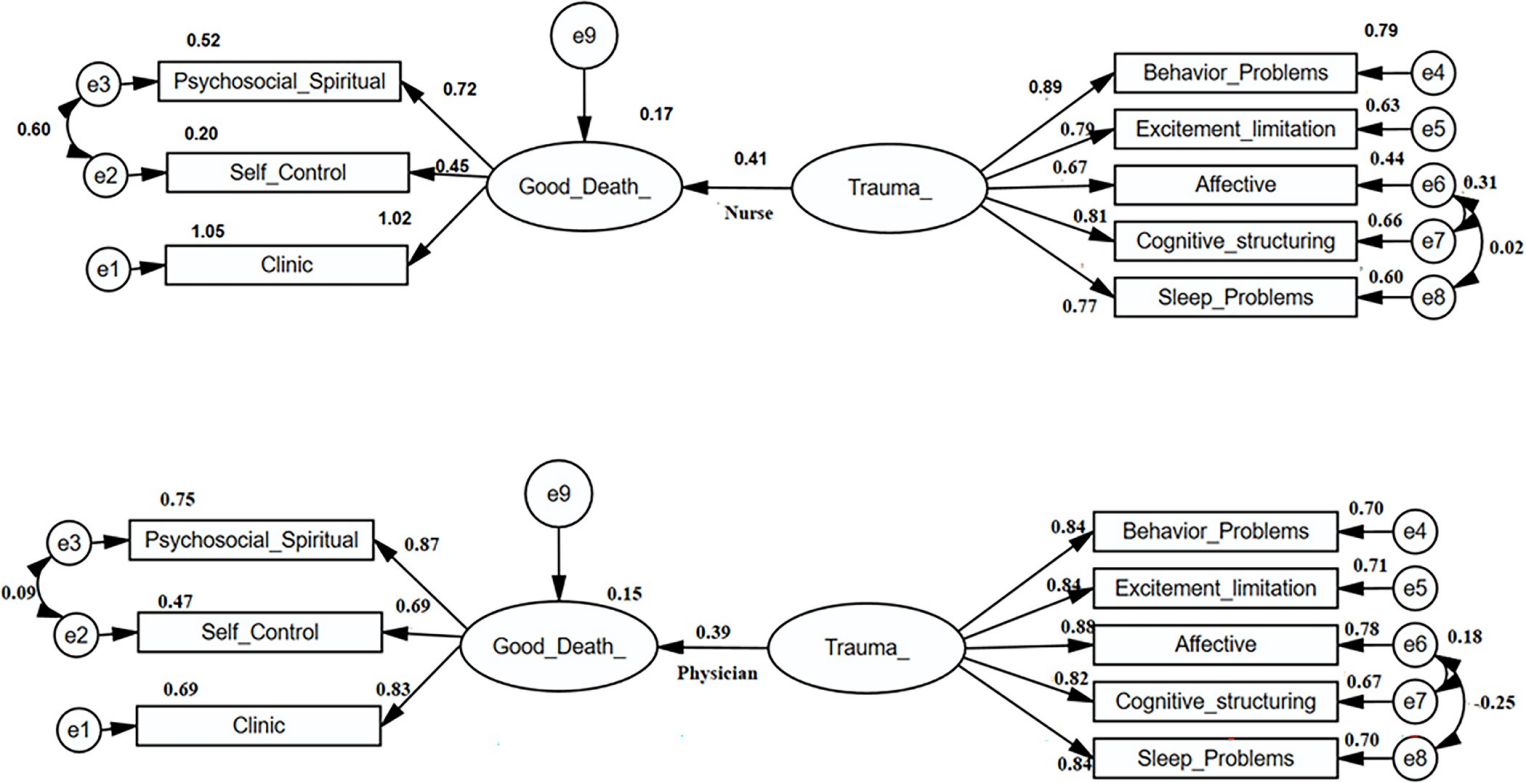
Multiple group analysis results of the relationship between the concept of good death and trauma level. The figure presents the multigroup analysis conducted to investigate whether the relationship between trauma and the concept of a good death differs between doctors and nurses. The analysis showed that the trauma level had a statistically significant effect on good death scores in both professions. However this effect was found to be slightly lower for physicians (an increase of 0.52 points in good death score for each one-point increase in trauma score), while it was slightly higher for nurses (an increase of 0.45 points in good death score for each one-point increase in trauma score).

Subsequently, a multiple group analysis was conducted to examine whether the relationship between trauma and a good death differed between physicians and nurses (Fig 2).

The differences between these two groups were found to be statistically significant indicating that the relationship between trauma and a good death varies by profession. The critical Z value used to test these differences was found to be 2.217, which is greater than the value of 1.96 indicating a statistical difference [21].

## Discussion

This study aims to determine the perception of a "good death" among physicians and nurses who experienced the earthquake and to understand the relationship between these perceptions and the levels of trauma caused by the earthquake. The majority of the participants were female (68.6%), aged between 25–34 years (58.8%), married (50%), had 0–5 years of work experience (58.6%), lived in a slightly damaged house due to the earthquake (49.1%), were still working in the earthquake zone (91.3%), and frequently thought about death. Only 18% of the participants had lost a first-degree relative.

According to the study’s findings, physicians and nurses are quite sensitive to the concept of a good death. These findings are consistent with similar studies in the literature [22–25]. The perception of a good death among healthcare professionals is important as it provides a positive foundation for sensitivity and effectiveness in patient care. This sensitivity can contribute to better care for patients in their final stages and increase the professional satisfaction of healthcare professionals.

The study found statistically significant differences between physicians and nurses in the subdimensions of the good death scale, namely "psychosocial spirituality," "personal control," and "clinical" scores (p<0.05). This difference indicates that nurses are more sensitive. Nurses play a crucial role in meeting the physical and psychosocial needs of patients, especially during end-of-life processes. Studies show that nurses are emotionally and psychologically more affected by the death of long-term care patients [26]. In this context, it can be said that nurses’ roles, which require more contact with patients and their relatives compared to physicians, affect their perception of a good death. The study’s findings are also consistent with the literature. In the study by Aksoy and Kaşıkçı, the participants’ average total score was found to be 56.84±7.51, the average score of the psychosocial and spiritual dimension was 30.64±4.10, the average score of the personal control subdimension was 10.10±1.91, and the average score of the clinical subdimension was 16.10±2.71. These results show that nurses have a high perception of a good death and consider death important in all dimensions [27]. In the study by Yıldız and colleagues that the participants had high scores in the psychosocial spiritual subdimension, personal control subdimension, and clinical subdimension scales. According to the professional group, the score obtained from the personal control subdimension was found to be significantly higher among nurses/health officers compared to physicians [25].

Studies have reported that frequent encounters with death can lead to various psychological and physical issues for nurses, including anxiety, depression, reduced job satisfaction, burnout, difficulties in patient communication, and depersonalization [28]. According to the data from this study, nurses are more sensitive to death-related issues and the frequency of thinking about death appears to be more pronounced in disaster situations. This context highlights the potential for disasters, such as earthquakes, to influence how nurses experience and cope with these challenges, underscoring the importance of support mechanisms to enhance their ability to manage these situations. In our study, high levels of trauma were found among both physicians and nurses. According to the obtained data, there were significant differences between physicians and nurses in terms of the subdimensions of the trauma scale, namely "emotional limitation," "emotional," "cognitive restructuring," and "sleep problems" scores (p<0.05). It was found that nurses were more affected than physicians. Our findings are consistent with the literature [29–32]. Even before the disaster, healthcare personnel experienced challenging times during the pandemic disaster. A review of the literature shows that the burnout levels of healthcare personnel significantly increased during the pandemic [33–41]. It can be said that the healthcare workers who experienced burnout during the pandemic and then went through the earthquake disaster experienced even higher levels of burnout. Additionally, it is important to consider that most of the participants in the study experienced the earthquake and have continued to work in the earthquake zone since the first day of the earthquake. This situation shows that the cumulative effect of successive challenging processes has a greater impact on the emotional and psychological health of healthcare professionals.

Researchers have comprehensively examined the psychological consequences of natural disasters [32,42–46]. The most common psychiatric diagnoses associated with disasters are major depressive episodes and post-traumatic stress disorder (PTSD) [47,48]. It is important for healthcare workers to receive psychological support during disaster times [49]. In the study by Li and colleagues, it was reported that nurses who experienced earthquakes had long-term psychological effects and did not receive psychological support [50]. Pourvakhshoori and colleagues also emphasized the importance of healthcare personnel knowing how to reduce their stress during disasters and having access to counseling services [51]. In particular, this study suggests that it is essential to remove healthcare workers who are earthquake victims from earthquake zones, ensure their own and their relatives’ safety, and prioritize the identification and support of their psychological and physiological needs.

An important finding of the study is the impact of this high level of trauma on the perception of a good death. The SEM analysis revealed a significant relationship between high trauma levels and the perception of a good death in both nurses and physicians. These results indicate that the trauma experienced by healthcare professionals is a significant factor in shaping their perception of a good death, especially influencing their perceptions related to death. According to the study by Keskin Kızıltepe and Karagöz, factors such as nurses’ personal-cultural characteristics, the clinic they work in, the duration of professional experience, having children, losing relatives, receiving education about death, frequently encountering death, fear of death, and the meaning they attribute to death affect nurses’ death anxiety, attitudes towards death, and perceptions [28]. In this study, it can be said that witnessing a large number of deaths, especially tragic ones, and the levels of trauma they experienced affect healthcare professionals’ perception of a good death.

## Limitation

The study could only be conducted in 4 of the 11 provinces affected by the earthquake due to administrative permissions and difficulties in reaching personnel. Information about psychological diagnoses, psychological treatment, and the use of psychological medication before the earthquake was not asked from the personnel. Data from participants who did not answer all the study questions for any reason were excluded from the study.

## Conclusion

Traumatic events such as earthquake disasters can profoundly affect the emotional states, trauma levels, and sensitivity to death of healthcare personnel. In this study, significant findings were obtained by examining the trauma levels and perceptions of a "good death" among physicians and nurses who experienced the earthquake.

The results of the study indicate that the trauma levels of healthcare professionals after major disasters like earthquakes have a significant impact on their perceptions of a good death. The fact that nurses have higher trauma levels compared to physicians and consequently exhibit higher perceptions of a good death is important for understanding the perceptual and emotional consequences of trauma.

These findings emphasize the need for healthcare systems to develop policies and support mechanisms aimed at addressing the emotional and psychological needs of healthcare workers and enhancing their resilience after disasters.

Additionally, this study provides a crucial foundation for future research to understand how trauma shapes the lives and job performance of healthcare workers in the long term. Such research will illuminate the comprehensive effects of trauma on the overall well-being of healthcare workers and contribute to the development of effective support strategies.

## Supporting information

S1 File Questionnaire. (DOCX)
