# Supplementary material for: Trauma levels and perspectives on dignified death among nurses and physicians who directly experienced the recent earthquake
Source: PLoS One. 2024 Oct 24;19(10):e0311184. doi: 10.1371/journal.pone.0311184 (PMC11501021; doi:10.1371/journal.pone.0311184)
Supplement: S1 File — (DOCX) [file pone.0311184.s001.docx]

**S1 Questionnaire. English version**

1. Your gender: ( )Female ( )Male
2. Your age: …………….
3. Your profession:
4. Your marital status:
5. How many children do you have?
6. Years of experience:
7. Your department/unit:
8. Your institution:
9. Are you still working in the earthquake-affected area?
10. Damage to your home during the earthquake:
11. Did you lose loved ones in the earthquake?
12. Whom did you lose?
13. Frequency of thoughts about death: (Constantly) (Sometimes) (Occasionally) (Never)

**Post-Earthquake Trauma Level Determination Scale**

|  |  | Strongly Disagree | Slightly Agree | Moderately Agree | Highly Agree | Completely Agree |
| --- | --- | --- | --- | --- | --- | --- |
|  | **Behavioral Problems** |  |  |  |  |  |
| 1 | I am experiencing loss of appetite. |  |  |  |  |  |
| 2 | I have become a more angry/irritable person. |  |  |  |  |  |
| 3 | I am having nightmares. |  |  |  |  |  |
| 4 | I cannot enter closed spaces due to the fear of an earthquake. |  |  |  |  |  |
|  | **Emotional Limitations** |  |  |  |  |  |
| 5 | I have lost my sense of security about the future. |  |  |  |  |  |
| 6 | It feels like life has no meaning anymore. |  |  |  |  |  |
| 7 | My desire to live has decreased after what I have experienced. |  |  |  |  |  |
| 8 | My regrets about what I have done in my life have increased after the earthquake. |  |  |  |  |  |
| 9 | I feel very helpless/powerless. |  |  |  |  |  |
|  | **Affective** |  |  |  |  |  |
| 10 | Needing help hurts my pride. |  |  |  |  |  |
| 11 | I have started to pay more attention to my behavior/relationships after the earthquake. |  |  |  |  |  |
| 12 | I appreciate the value of my life more. |  |  |  |  |  |
| 13 | I have become very emotional/I cry for no reason. |  |  |  |  |  |
|  | **Cognitive Restructuring** |  |  |  |  |  |
| 14 | I worry about my children/parents/friends. |  |  |  |  |  |
| 15 | I am anxious with the thought that an earthquake might happen at any moment. |  |  |  |  |  |
| 16 | Images of the earthquake come to my mind. |  |  |  |  |  |
| 17 | I am worried about the future. |  |  |  |  |  |
|  | **Sleep Problems** |  |  |  |  |  |
| 18 | I wake up suddenly from sleep. |  |  |  |  |  |
| 19 | I have difficulty falling asleep. |  |  |  |  |  |
| 20 | I sleep less. |  |  |  |  |  |

**Good Death Scale**

|  | 1=Not important | 2=Somewhat important | 3=Important | 4=Very important |
| --- | --- | --- | --- | --- |
| Sudden and largely painless death |  |  |  |  |
| Short duration of death |  |  |  |  |
| Sudden and unexpected death |  |  |  |  |
| Family and doctors observing the person's wishes |  |  |  |  |
| Death occurring naturally without technical devices |  |  |  |  |
| Peaceful death |  |  |  |  |
| Presence of loved ones |  |  |  |  |
| Meeting the person's spiritual needs |  |  |  |  |
| Acceptance of death by the person |  |  |  |  |
| Having a chance to complete important duties |  |  |  |  |
| Having an opportunity to say goodbye |  |  |  |  |
| Being at home |  |  |  |  |
| Living until very important events occur |  |  |  |  |
| Death occurring during sleep |  |  |  |  |
| Having mental functions intact until death |  |  |  |  |
| Having control over bodily functions until death |  |  |  |  |
| Having communication abilities until death |  |  |  |  |
